# Temporal and Spatial Analysis of Rabies Virus Lineages in South Africa

**DOI:** 10.3390/v17030340

**Published:** 2025-02-28

**Authors:** Natalie Viljoen, Claude Sabeta, Wanda Markotter, Jacqueline Weyer

**Affiliations:** 1 Centre for Viral Zoonoses, Department of Medical Virology, University of Pretoria, Pretoria 0001, South Africa; natalie.viljoen@uct.ac.za (N.V.); wanda.markotter@up.ac.za (W.M.); 2 Centre for Emerging Zoonotic and Parasitic Diseases, The National Institute for Communicable Diseases of the National Health Laboratory Service, Johannesburg 2131, South Africa; 3 Department of Veterinary Tropical Diseases, Faculty of Veterinary Science, University of Pretoria, Pretoria 0001, South Africa; claude.sabeta@up.ac.za; 4 Department of Microbiology and Infectious Diseases, Faculty of Health Sciences, University of Witwatersrand, Johannesburg 2000, South Africa

**Keywords:** rabies, rabies virus, surveillance, distribution, wildlife, domestic dogs, South Africa

## Abstract

Rabies virus (RABV; species *Lyssavirus rabies*) causes rabies, a disease of the central nervous system that invariably results in the death of the host. In South Africa, studies have indicated that RABV is maintained by animal species that include four wildlife carnivore species—the black-backed jackal (*Canis mesomelas*), bat-eared fox (*Otocyon megalotis*), yellow mongoose (*Cynictis penicillata*), and aardwolf (*Proteles cristatus*)—and domestic dogs (*Canis lupus familiaris*). The complex natural ecology holds significant implications for the control and elimination of rabies. In this study, confirmed animal rabies case data, including geospatial features, were analyzed for 12,879 laboratory-confirmed animal cases reported on a database managed by the Department of Agriculture, Land Reform and Rural Development (DALRRD). Sequence data generated from animal rabies cases in South Africa were also analyzed, which included 1374 cytoplasmic domain of the glycoprotein and the G-L intergenic sequences using maximum likelihood (ML) and Bayesian inference. The analysis provides insights into the transmission dynamics involving several wildlife species and domestic dogs in South Africa. This information is crucial for the strategic planning for rabies control and elimination programs, and particularly in understanding the interlinked nature of some lineages and the importance of the cross-border spread of rabies. This analysis provided an improved understanding of the distribution of the RABV lineages in South Africa and identified areas that can be targeted for rabies control strategies to limit future spread of RABV, which is important due to the limited available resources that must be carefully managed to allow optimal control.

## 1. Introduction

In 2015, the United Against Rabies collaboration was established between the World Health Organization (WHO), the Food and Agriculture Organization of the United Nations (FAO), the World Organization for Animal Health (WOAH), and the Global Alliance for Rabies Control (GARC) with the goal to eliminate dog-mediated human rabies by 2030. The three-phase plan to accomplish this goal includes (1) the effective use of vaccines, medicines, tools, and technologies to prevent the transmission of dog rabies and reduce human deaths due to rabies; (2) the use of high-quality data and evidence-based guidance to measure the impact of existing measures and inform policy decisions; and (3) the sustained commitment and provision of resources by engaging with multiple stakeholders [1]. Understanding rabies epidemiology and natural ecological cycles is imperative to enable the implementation of effective, targeted rabies control and elimination strategies. The most cost-effective rabies control strategy to eliminate dog-mediated human rabies is the vaccination of domestic dogs. The WHO recommends vaccination coverage of at least 70% against rabies, a target generated through models and predictions, and sufficient to prevent or control an outbreak in domestic dogs [2]. It is important to acknowledge that environmental and population-based factors may influence the vaccination coverage required in a particular region.

In Africa, reports of rabies-like illness in dogs and humans preceded the first confirmed outbreak of rabies reported in the Eastern Cape, South Africa in 1893 [3]. In southern Africa, two rabies virus (RABV; species *Lyssavirus rabies*) variants have been identified, the canid and mongoose variant [4,5,6,7]. In South Africa, the canid variant can be maintained independently or interdependently by black-backed jackals, bat-eared foxes, aardwolves, or domestic dogs [6,7,8,9,10,11], whereas the mongoose variant is primarily maintained in the yellow mongoose with spillover to other *Herpestidae* and non-*Herpestidae* species [5,12,13]. Spillover into the host range of the other variant (for example, spillover of the canid variant to mongooses or vice versa) can occur [14]; however, this appears to frequently result in dead-end infections [13]. The mongoose and canid variants evolved independently, with the presence of the mongoose variant likely predating the introduction of the present canid variant into southern Africa [15].

In 1928, after 34 years without any reports of rabies in South Africa, the disease was confirmed in two children who were bitten by a yellow mongoose (*Cynictis penicillata*) in Wolmaranstad [3], which led to the suspicion that a viverrid form (mongoose variant) of rabies was present in South Africa. The yellow mongoose was later demonstrated to be susceptible to RABV infection with both the mongoose and canid variant RABV, but in contrast to infection with the canid variant, a higher mortality rate and more frequent virus shedding was observed when yellow mongooses were infected with the mongoose variant [16]. In addition, the mongoose and canid variants were demonstrated to be genetically distinct [5]. The canid variant is thought to have been introduced into South Africa several times via the importation of infected animals and from neighboring countries [3]. Two of the most significant introductions of RABV into South Africa occurred in 1961 from southern Mozambique into northern KwaZulu Natal, which resulted in an unprecedented outbreak in domestic dogs (*Canis lupus familiaris*) and extended southward across the province. This was followed by reintroduction from Mozambique in 1976, which resulted in the spread of RABV to the Transkei and Ciskei territories (today part of the Eastern Cape province) by 1987 and the early 1990s, respectively [3].

In South Africa, there are five recognized rabies maintenance hosts, which include domestic dogs and four wildlife hosts, namely the bat-eared fox (*Otocyon megalotis*), black-backed jackal (*Canis mesomelas*), aardwolf (*Proteles cristatus*), and yellow mongoose [5,7,17,18]. Spatiotemporal analysis of animal rabies cases using passive surveillance data from the Department of Agriculture, Land Reform and Rural Development (DALRRD), South Africa, which included confirmed rabies cases between 1993 and 2019, revealed that wildlife species formed part of 9 of 13 identified rabies disease clusters in animals [19]. In a different study, using laboratory testing data, including positive and negative samples from the two animal reference laboratories in South Africa between 1998 and 2019, disease clusters for specific groups of animals were identified and included five disease clusters in domestic animals, three disease clusters in wildlife excluding mongooses, two disease clusters in mongooses, and five disease clusters in livestock [20]. Importantly, geographic overlap between disease clusters identified for different groups of animals was demonstrated. However, the disease clusters were not always identified for the same timeframes, which suggested that one maintenance host can contribute to disease clusters in a region and that the dominant maintenance host may change with time [20]. This has important implications for rabies control; however, since domestic dogs are the most likely species to come into contact with humans, it is plausible that human cases can be significantly reduced by the successful implementation and sustained vaccination of domestic dog populations.

In this study, data for laboratory-confirmed animal rabies cases, geospatial features, and publicly available sequence data for South Africa were analyzed. RABV lineages were identified and, for each lineage, the host and spatiotemporal characteristics evaluated.

## 2. Materials and Methods

### 2.1. Analysis of Animal Rabies Case Data

Approval for analysis of the data in this study was obtained in terms of Section 20 of the Animal Diseases Act, 1984 (Act no 35 of 1984) under the reference number 12/11/1/1/25 (6366 JPC). All verified laboratory-confirmed animal rabies cases in South Africa are reported to DALRRD and are available in a publicly shared database for animal diseases (http://webapps1.daff.gov.za/VetWeb/dieaseDatabase.do, accessed on 24 May 2022). Data was extracted for the period January 1993 to December 2021. Data curation was performed to confirm its correctness and subsequent analysis by inspecting all columns to ensure that the correct case numbers were reported for each event. For temporal analysis, all data were sorted based on the locality of origin (reporting province) and grouped separately for each province and the Kruger National Park, and subsequently sorted by date and species, and the number of rabies cases per province/park per year was collated. For district-level analysis, data from January 2010 to December 2021 were analyzed by first assigning the correct district to each case according to the current division of districts and metropolitan municipalities, and then sorting cases based on the reporting district and species. The number of cases in each province for each district per species was collated. To provide improved resolution, district-level analysis was performed from January 2010 to December 2021. Where changes were identified, more focused analysis at municipal level was performed. Graphs were constructed using Microsoft Excel V2501 (Microsoft Corporation).

### 2.2. Analysis of Rabies Sequence Data

Sequence data for RABV from South Africa were obtained from GenBank and aligned using Muscle [21] in Molecular Evolutionary Genetics Analysis (MEGA) V11.0.11 [22]. Aligned sequences were trimmed to include the 592-nucleotide region that encodes the cytoplasmic domain of the glycoprotein and the G-L intergenic sequence. Subsequent analyses were performed on 1374 sequences after the exclusion of sequence data with insufficient coverage, sequence data from human rabies cases, and duplicate sequences identified by the unique laboratory identification number. All phylogenetic analyses were performed on the Cyber Infrastructure for Phylogenetic RESearch (CIPRES) server [23] on the Extreme Science and Engineering Discovery Environment (XSEDE) [24]. A maximum likelihood (ML) tree was constructed using IQ-Tree V2.1.2 [25] using the general time reversible (GTR) substitution model with 1000 bootstrap replicates. Phylogenetic trees were rendered and annotated using Interactive Tree of Life (ITOL) [26]. The ML tree was inspected and two datasets were prepared, one containing all mongoose variant RABV sequences, and the other containing all canid variant RABV sequences. The overall mean distance (OMD) was determined for the RABV canid and mongoose variant in MEGA V11.0.11 [22] using the p-distance method to calculate the OMD with 1000 bootstrap replicates, considering transitions and transversions with gamma-distributed rates among sites and pairwise deletions, and was converted to percentage similarity by 100−(OMD×100).

RABV lineages were identified on the phylogenetic tree rendered for the complete South African RABV dataset. For each lineage, the percentage contribution of sequence data from each of the known maintenance hosts was determined. Based on the host distribution, each lineage appeared to have one or two dominant maintenance hosts each and was subsequently named based on the dominant maintenance host(s). To confirm lineage assignment using Bayesian and ML inference, RABV sequences for all lineages with the same dominant maintenance hosts were grouped into separate datasets, which included the mongoose (MON), domestic dog (DD), dog-jackal (BBJ-DD), and bat-eared fox (BEF) datasets. The best-fit model of nucleotide substitution was determined for each dataset using the Bayesian information criterion in JModelTest2 V2.1.10 [27] and applied using Bayesian Evolutionary Analysis Sampling Trees (BEAST) V2.7.3 [28]. An underlying coalescent process with a constant population size was assumed. Markov chain Monte Carlo (MCMC) chains of 50–100 million generations were used; however, for the dog-jackal dataset adequate effective sample size (ESS) values could not be obtained and the analysis was repeated using the BModelTest package in BEAST V2.7.3 [28] with MCMC chains of 50 million generations. Tracer V1.7.2 [29] was used to visualize and analyze MCMC trace files and TreeAnnotator V2.6.6 [28] was used to identify the best-fit tree with a burn-in of 10%. ML inference was performed using the IQ-Tree webserver available at http://iqtree.cibiv.univie.ac.at/ (accessed on 29 March 2023) using the GTR G+I substitution model and 1000 ultrafast bootstrap analysis. Interactive Tree of Life (ITOL) [26] was used to render and annotate phylogenetic trees. All phylogenetic trees were rooted using an RABV sequence obtained from a jackal in northern Namibia that does not form part of any of the RABV lineages in South Africa (GenBank accession number: JX473839.1) and were in agreement.

To assess the distribution of rabies cases for each identified lineage, the available global positioning system (GPS) coordinates were plotted using Quantum Geographic Information System (QGIS) [30]. The minimum distribution of each lineage was determined by connecting cases from districts with evidence of rabies cases with time (repeated evidence of cases linked to a specific lineage from the same district); however, cases reported during outbreaks resulting from one or more imported cases were excluded. It is important to note that autochthonous transmission of RABV after introduction into a non-endemic area can result in RABV becoming established in the area; however, in our analyses, such an area would only be included in the minimum distribution mapping if evidence to support the reoccurrence of cases in the area after the outbreak was available. Maps were constructed and layered with the national, provincial, district, and municipal borders in South Africa available at https://data.humdata.org/dataset/cod-ab-zaf? (accessed on 13 April 2023).

In addition, representative sequences from each identified lineage in South Africa and from neighboring countries were analyzed to identify lineages across national borders, which were identified using BEAST V2.7.3 [28] with the BModelTest package with MCMC chains of 50 million generations, and an underlying coalescent process with a constant population size was assumed. Tracer V1.7.2 [29] was used to visualize and analyze MCMC trace files and TreeAnnotator V2.6.6 [28] was used to identify the best-fit tree with a burn-in of 10%. Interactive Tree of Life (ITOL) [26] was used to render and annotate phylogenetic trees.

## 3. Results

### 3.1. Number and Distribution of Laboratory-Confirmed Animal Rabies Cases Based on Surveillance Data

**Figure 1:**
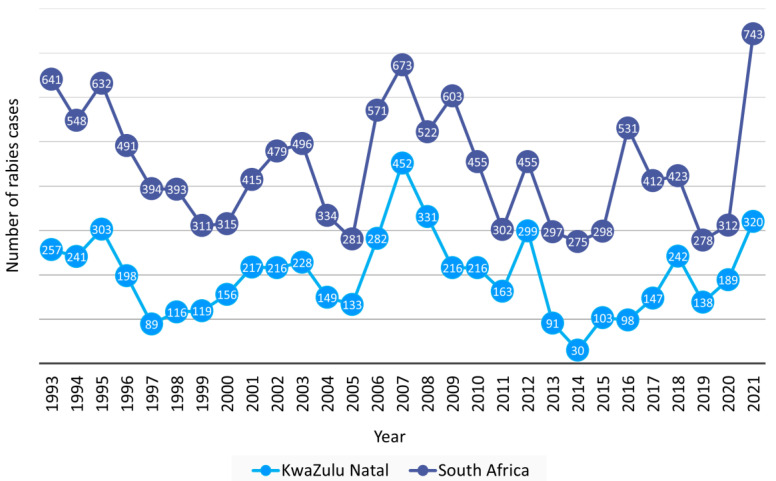
Laboratory-confirmed animal rabies cases reported from the KwaZulu Natal province and all provinces in South Africa, 1993–2021. The number of cases reported in the KwaZulu Natal province per year is indicated in light blue and the total number of animal cases reported from South Africa, including cases from the KwaZulu Natal province is indicated in dark blue.

**Figure 2:**
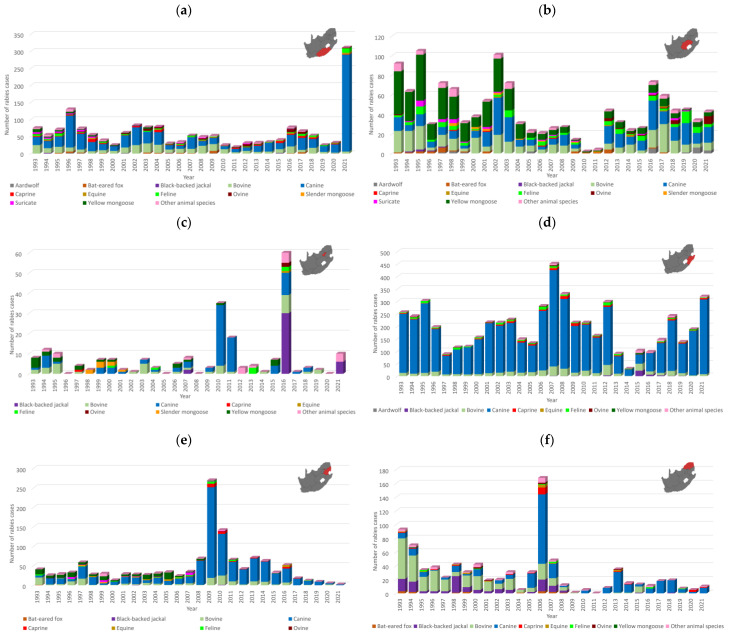

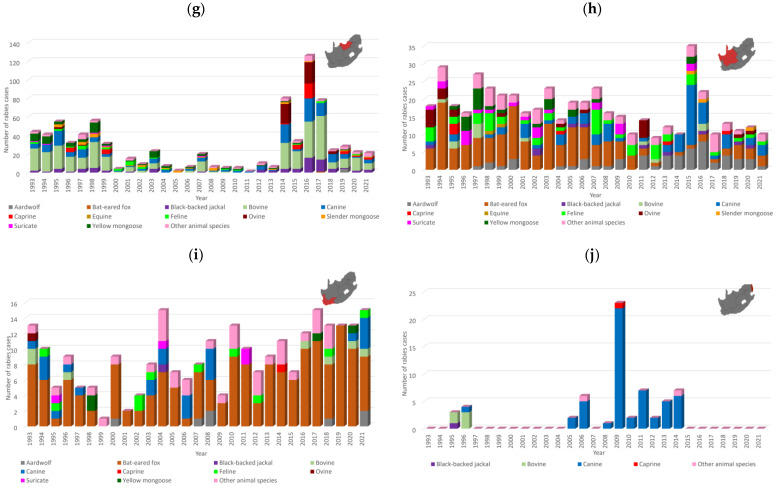
Temporal analysis of animal rabies cases per province and the Kruger National Park, 1993–2021. The number of rabies cases reported every year for selected animal species per province is indicated for the (**a**) Eastern Cape, (**b**) Free State, (**c**) Gauteng, (**d**) KwaZulu Natal, (**e**) Mpumalanga, (**f**) Limpopo, (**g**) North West, (**h**) Northern Cape and (**i**) Western Cape provinces, and the (**j**) Kruger National Park. The region of interest is shown in red on the map. Based on the available data, animal species that comprise at least 0.5% of the total number of rabies cases reported from 1993 to 2021 as reported in Table S1 are indicated, whereas the cases from all other animal species are represented collectively. It is important to note that there were years in which no cases were reported for some animal species in the represented geographical areas.

A total of 12,879 laboratory-confirmed animal rabies cases were reported to DALRRD over 29 years, 1993–2021, with an average of 444 and a range of 275–743 verified cases per year (Figure 1). KwaZulu Natal province had the highest number of animal rabies cases and reported 44.6% (5739/12,879) of cases in South Africa. Subsequently, the trend of animal rabies cases in South Africa was largely determined by cases reported from the KwaZulu Natal province (Figure 1). A surge of animal rabies cases occurred in 2021 in domestic dogs in the Eastern Cape and KwaZulu Natal provinces (Figure 2a,d) and resulted in the highest number of recorded animal rabies cases in South Africa since 1928. The distribution of cases in these two outbreaks appeared to be highly clustered in and around the metropolitan municipalities and correlated with an increase in human rabies cases reported from the Eastern Cape and KwaZulu Natal provinces [31]. The cases reported from these two provinces accounted for 84.9% (631/743) of cases reported in 2021; however, it is important to note that a higher number of cases was reported for the second part of 2021 for the Nelson Mandela Bay metropolitan municipality in the Eastern Cape elsewhere [32].

The distribution of rabies cases in animal species from 1993 to 2021 underscored the role of the domestic dog (61.5%, 7914/12,879), black-backed jackal (2.3%, 301/12,879), bat-eared fox (3.2%, 409/12,879), and yellow mongoose (5.2%, 675/12,879) as maintenance hosts. Since the aardwolf (0.76%, 98/12,879) is an emerging maintenance host, the number of reported cases is lower than that reported for the other maintenance hosts. The distribution of rabies cases suggested that considerable spillover occurred to cattle (15.4%, 1985/12,879), domestic cats (3.2%, 413/12,879), goats (2.2%, 277/12,879), and sheep (1.3%, 171/12,879), with limited spillover in a variety of other animal species (4.9%, 636/12,879) (Table S1). Temporal analysis suggested that spillover infections were associated with an increase in cases in the dominant maintenance host(s) within an area and the animal species affected differed from region to region likely due to the type of interactions with an infected maintenance host and the animal rearing activities and practices in the area (Figure 2a–i). Livestock and humans are considered to be dead-end hosts and do not play a role in the maintenance of RABV since transmission from these species is exceedingly rare.

**Figure 3:**
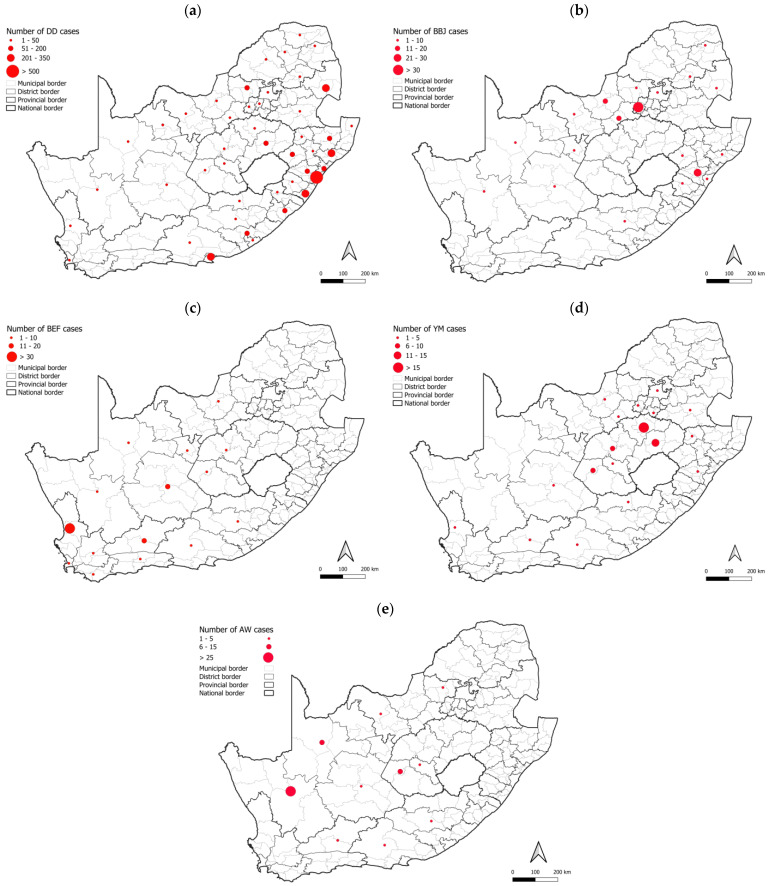
Distribution of cases in maintenance hosts per district, 2010–2021. The number of rabies cases reported per district or metropolitan municipality is illustrated for (**a**) domestic dogs, (**b**) black-backed jackals, (**c**) bat-eared foxes, (**d**) yellow mongooses, and (**e**) aardwolves. The scale for each animal species is indicated in the top left corner to demonstrate the range that each size circle represents. Selected districts or metropolitan municipalities with a high number of cases are indicated. The maps were constructed using QGIS and layered with the national, provincial, district, and municipal borders in South Africa available at https://data.humdata.org/dataset/cod-ab-zaf? (accessed on 13 April 2023).

The distribution of rabies cases in maintenance hosts in South Africa is provided for each district from 2010 to 2021 in Figure 3. Rabies cases were reported in domestic dogs throughout the country; however, a high number of cases were reported from large parts of the KwaZulu Natal province, particularly the eThekwini metropolitan municipality, the Nelson Mandela metropolitan municipality in the Eastern Cape, and the Ehlanzeni district in Mpumalanga province (Figure 3a). Another noteworthy area was the Thabo Mofutsanyana district in the Free State, particularly the Dihlabeng and Mantsopa municipalities, which share a border with Lesotho and reported 73.5% (61/83) of cases from this district.

In contrast to the expected distribution of rabies cases in the black-backed jackal, two hotspots were identified in areas that historically reported sporadic or no cases in the black-backed jackal, which included the West Rand district in Gauteng and uMgungundlovu district in KwaZulu Natal. In addition, Limpopo province reported a low number of rabies cases in the black-backed jackal (Figure 3b), which has long been considered to play an important role in maintaining RABV in this province due to the availability of farming areas conducive for the proliferation of jackal populations. However, a higher number of black-backed jackal rabies cases were reported in Limpopo province between 2013 and 2018 elsewhere [33]. Despite the high number of cases in domestic dogs in KwaZulu Natal province, limited spillover is typically reported to wildlife (Figure 2d). Investigation of the factors driving the expansion of RABV in wildlife is required because rabies cases have been reported in black-backed jackals in or around these two regions in subsequent years (Figure 2c,d), suggesting continued expansion of RABV-infected black-backed jackal populations, which may result in spillover to other animal species and human exposure to RABV.

Rabies cases in the bat-eared fox outnumbered cases in all other animal species, including domestic dogs, in the Northern and Western Cape provinces and fluctuated significantly with time with limited evidence of spillover to other species (Figure 2h,i). However, it is important to note that a shift in reporting occurred for the Northern Cape province after 2010, with the number of aardwolf cases outnumbering the number of cases reported in bat-eared foxes, which was reported at the lowest level since 1993 and has remained low (Figure 2h). The majority of bat-eared fox cases were reported from the West Coast district in the Western Cape province (Figure 3c) and were primarily reported from the Saldanha Bay and Bergrivier municipalities, which represented 70.5% (55/78) of cases reported from this district. Since 2013, a significant increase in aardwolf rabies cases has been reported, with the majority of cases reported from the Namakwa district in the Northern Cape, particularly the Nama Khoi and Khai-ma municipalities, and a fairly localized area in the Free State partly in the Mohokare municipality in the Xhariep district and Mangaung metropolitan municipality (Figure 3e). Other than these two regions, aardwolf cases were sporadically reported. Rabies cases in the yellow mongoose were primarily reported from the Free State province, particularly the Fezile Dabi and Thabo Mofutsanyana districts (Figure 3d); however, the low number of cases reported from the Gert Sibande district in Mpumalanga province, where a mongoose variant was previously demonstrated to be present [34], is curious (Figure 2e) and may, in part, be due to the destruction of the habitat of the yellow mongoose. This requires further investigation.

### 3.2. RABV Lineages and Distribution Based on Geospatial and Molecular Epidemiological Data

In this study, sequence data for the cytoplasmic domain of the glycoprotein and the G-L intergenic region were analyzed because the majority of RABV sequence data available for South Africa is based on the cytoplasmic domain of the glycoprotein and the G-L intergenic region and most regions are well-represented in this dataset; however, some regions and animal species are underrepresented. In addition, a previous study demonstrated that the same branching was observed for phylogenetic trees based on the nucleoprotein gene and the G-L intergenic region [35]. The branch lengths of an ML tree inferred from RABV G-L intergenic sequence data suggested a low degree of genetic divergence among RABV canid variant sequences compared to the mongoose variant sequences from South Africa, an observation consistent with previous findings [8,12]. The canid variant sequence data analyzed were 96.97% similar whereas the mongoose variant sequence data were 87.25% similar.

**Figure 4:**
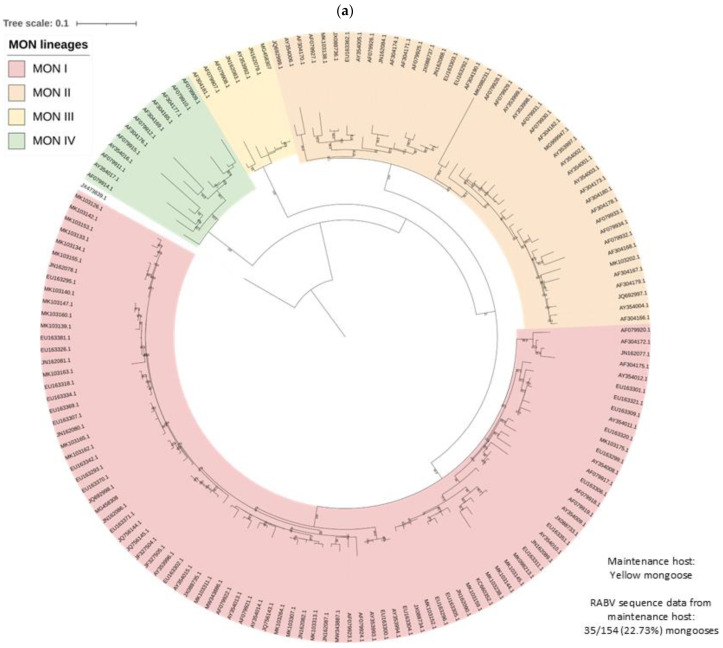

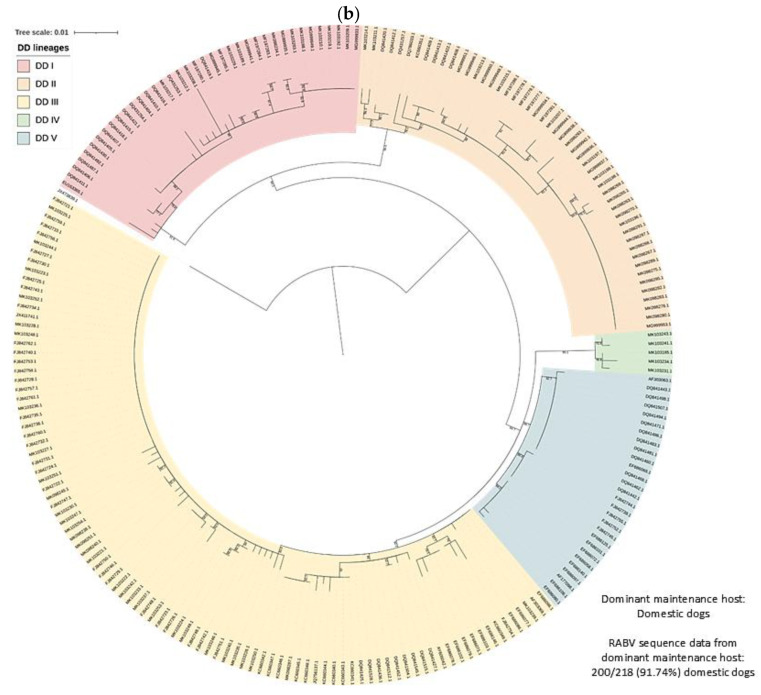

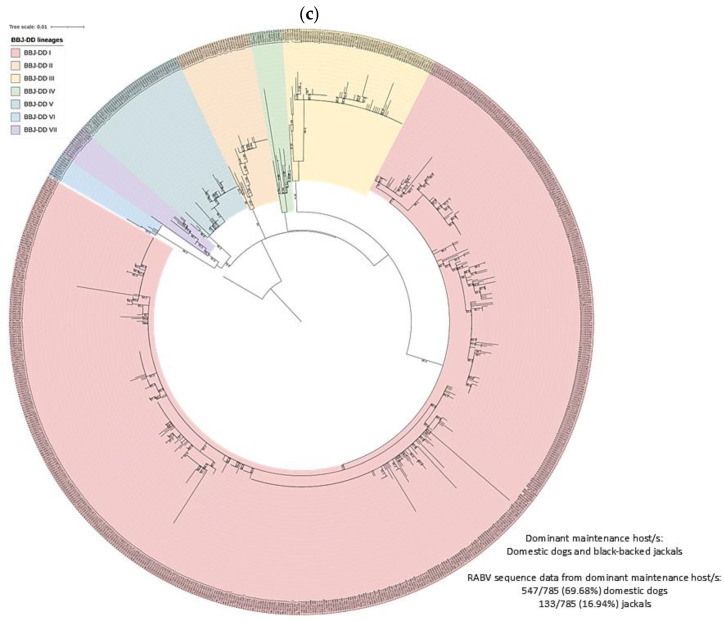

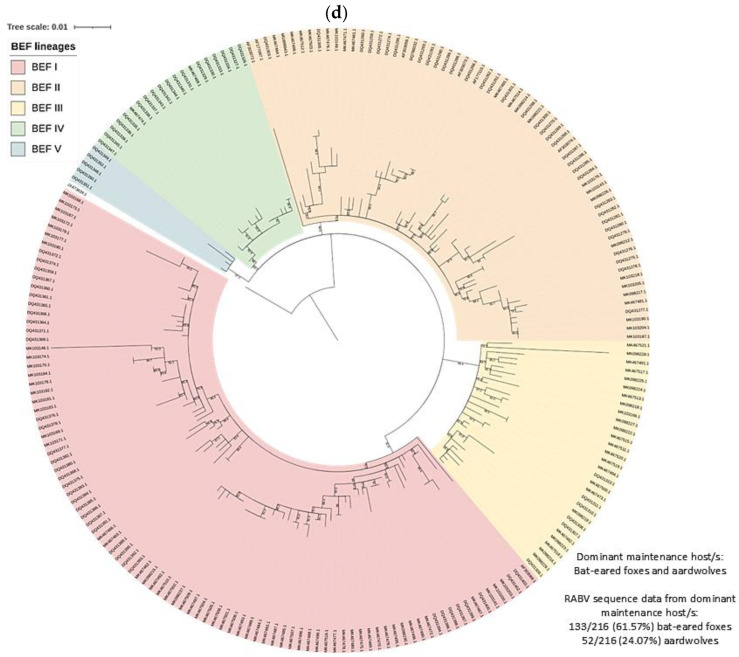
RABV lineages in South Africa grouped based on the dominant maintenance host(s). ML phylogenetic trees inferred from cytoplasmic domain of the glycoprotein and G-L intergenic sequence data are provided. An ML tree with the complete South African dataset is provided (right) and an ML tree for the (**a**) MON, (**b**) DD, (**c**) BBJ-DD, and (**d**) BEF datasets (left) demonstrating the lineages identified for each and the relative position within the complete ML tree.

RABV lineages identified using ML and Bayesian inference were in agreement. Outlier sequences were identified and may represent imported cases or lineages that are not well represented by existing sequence data. All lineages were supported by bootstrap values higher than 70 on the ML phylogenetic tree, except for the MON III lineage. The maintenance host(s) for each lineage was identified and subsequently lineages were grouped and analyzed accordingly (Figure 4 and Figure S1; Phylotree S1–S4).

To determine the geographical distribution of rabies cases within each identified RABV lineage, the reported coordinates, or the closest city/town/farm in the absence of coordinates, of all cases were mapped. The minimum distribution was mapped and provided an estimated distribution of the lineage only considering cases from districts or metropolitan municipalities with repeated evidence of rabies cases, except cases reported during outbreaks due to the importation of RABV (Table S2).

**Figure 5:**
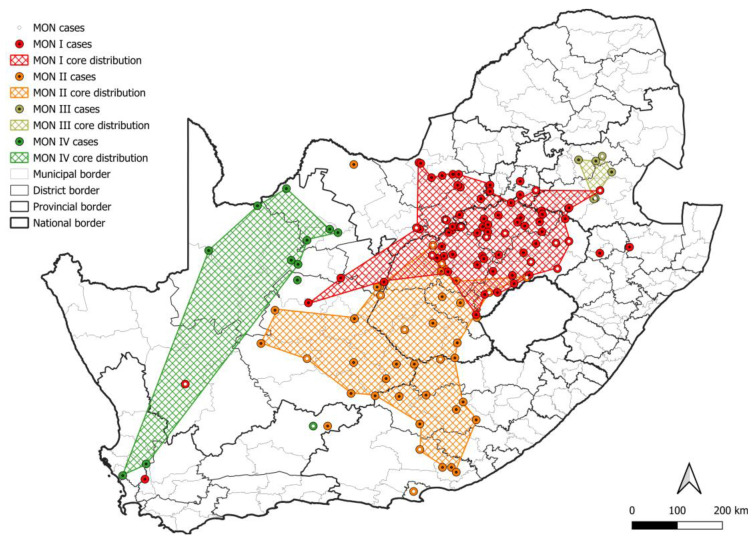
The distribution of mongoose RABV lineages in South Africa. The cases that belong to each MON lineage are plotted. Cases reported in the dominant maintenance host are indicated; however, since the mongoose species is frequently not specified, all mongoose cases are indicated. The map was constructed using QGIS and layered with the national, provincial, district, and municipal borders in South Africa available at https://data.humdata.org/dataset/cod-ab-zaf? (accessed on 13 April 2023).

At least four mongoose lineages, designated MON I-IV, were identified (Figure 4a). All MON lineages were supported by bootstrap values higher than 99.5, except MON III, which had a bootstrap value of 56.5 but formed a distinct ground on the phylogenetic tree, and these rabies cases appeared to be restricted to the central and eastern parts of Mpumalanga (Figure 5; Table S1). Historically, the mongoose variant is well accepted to be primarily distributed across the central plateau and, while the two lineages, MON I and MON II, that represented the majority of rabies cases for which sequence data was available were distributed across the central plateau, mapping of the MON lineages suggested a significantly wider dispersion of cases (Figure 5; Table S2); however, isolated cases may have been associated with the movement of animals. MON I and MON II have overlapping distribution and appeared to represent sister groups that evolved from a common ancestor. For MON I, sequence data linked isolated cases that were geographically separated from the minimum distribution in wildlife in the Northern Cape, KwaZulu Natal, and Western Cape provinces and domestic dogs in Gauteng and the Northern Cape provinces. For MON II, limited sequence data for some regions, including the Nelson Mandela Bay metropolitan municipality in the Eastern Cape and Central Karoo district in the Western Cape, may have resulted in an underestimation of the distribution. MON III and MON IV appeared to be restricted to parts of the Mpumalanga province, and Northern and Western Cape provinces, respectively; however, for MON IV, large distances exist between mapped cases, and the majority of cases were identified in wildlife carnivores with only two cases in domestic dogs in the John Taolo Gaetsewe district, Northern Cape province.

**Figure 6:**
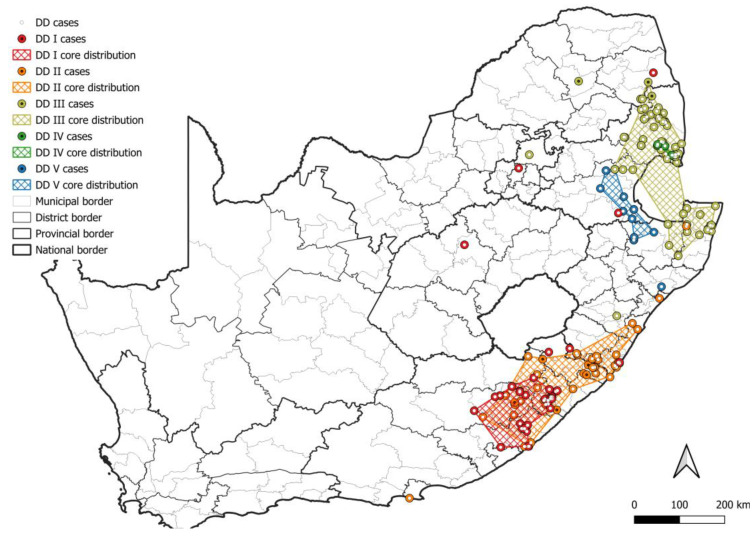
The distribution of RABV lineages exclusively maintained by domestic dogs in South Africa. The cases that belong to each DD lineage are plotted. Cases reported in the dominant maintenance host are indicated. The map was constructed using QGIS and layered with the national, provincial, district, and municipal borders in South Africa available at https://data.humdata.org/dataset/cod-ab-zaf? (accessed on 13 April 2023).

In South Africa, the canid variant is maintained (1) exclusively by domestic dogs with little to no demonstrable involvement from wildlife hosts, (2) by an interplay between domestic dogs and black-backed jackals, (3) by bat-eared foxes with little to no involvement from domestic dogs, black-backed jackals, or aardwolves, (4) by an interplay between bat-eared foxes and aardwolves, or (5) exclusively by aardwolves. RABV lineages exclusively maintained by domestic dogs were restricted to the eastern side of the country (Figure 6), and corresponded to regions reporting the highest number of cases in domestic dogs except for the metropolitan municipalities in the Eastern Cape that reported exceptionally high numbers of cases in domestic dogs (Figure 3a). However, sequence data for the metropolitan municipalities in the Eastern Cape are limited or unavailable and are required to determine whether a separate RABV lineage is present and to confirm if RABV is exclusively maintained by domestic dogs.

At least five lineages that appeared to be exclusively maintained by domestic dogs (DD I–V) were identified (Figure 6), of which at least two were previously described [8], DD I and DD II. All DD lineages were supported by bootstrap values higher than 80.0, except DD V with a bootstrap value of 74.9 (Table S1). Two sister groups that each appeared to have evolved from a common ancestor were identified and have an overlapping distribution, which consisted of the DD I and DD II lineages, and DD III, DD IV, and DD V lineages, respectively. Interestingly, two black-backed jackal cases in the Mopani district and one case in the Capricorn district in Limpopo were linked to DD III, which suggested that these cases likely represented spillover after the importation of an infected animal, likely a domestic dog. This highlights the importance of controlling RABV in domestic dogs to limit spillover to wildlife hosts and the potential consequences of the transportation of infected animals.

**Figure 7:**
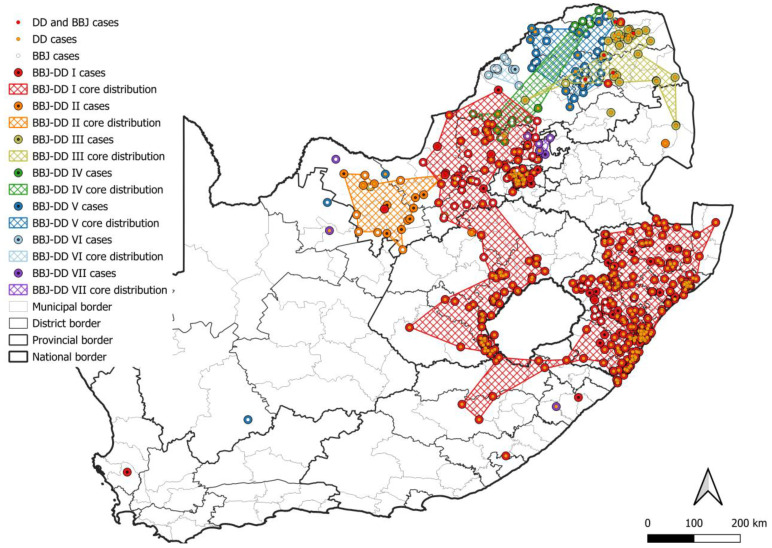
The distribution of RABV lineages maintained by an interplay between domestic dogs and black-backed jackals in South Africa. The cases that belong to each BBJ-DD lineage were plotted. Cases reported in the dominant maintenance hosts were indicated. The map was constructed using QGIS and layered with the national, provincial, district, and municipal borders in South Africa available at https://data.humdata.org/dataset/cod-ab-zaf? (accessed on 13 April 2023).

At least seven lineages that appeared to be maintained by an interplay between domestic dogs and black-backed jackals (BBJ-DD I–VII) were identified (Figure 7). All BBJ-DD lineages were supported by bootstrap values higher than 85.0 (Table S1). BBJ-DD I spanned parts of seven of the nine provinces in South Africa with the exception of the Northern and Western Cape provinces. Although rabies cases from Gauteng province formed part of this lineage, almost all cases were linked to outbreaks due to the introduction of RABV from other provinces. Repeated evidence of cases outside outbreak periods was only identified in the City of Tshwane, with sporadic cases from other districts and metropolitan municipalities, and only the City of Tshwane was therefore included in the minimum distribution of this lineage. Phylogenetic analyses suggested that cases from the other districts and metropolitan municipalities were imported and likely due to the movement of animals over long distances (for example, from KwaZulu Natal and the Free State provinces) or due to the natural movement of animals (for example, from the North West province due to the natural movement of RABV-infected black-backed jackals). Due to limited sequence data, repeated representation of this lineage could not be demonstrated for parts of the Eastern Cape (Alfred Nzo and OR Tambo districts) and Free State (Fezile Dabi district) provinces; however, the distribution of cases suggested that these districts likely form part of this lineage and they were therefore included in the minimum distribution (Table S2). The black-backed jackal appeared to be the primary maintenance host in two lineages, BBJ-DD II and BBJ-DD VI, with little or no evidence of the involvement of domestic dogs, which was previously described [10,11,35]. BBJ-DD II appeared to be confined to the western parts of the North West province and consisted almost exclusively of sequences from rabies cases in the black-backed jackal and domestic cattle with sporadic cases in domestic dogs. BBJ-DD VI appeared to be mostly confined to the Waterberg district in Limpopo province, with sporadic cases reported from the Vhembe district, and consisted exclusively of black-backed jackal and bat-eared fox cases dating from 2000 to 2016, which suggested that black-backed jackals and/or bat-eared foxes likely maintained RABV over extended periods with no demonstrable involvement of domestic dogs. Interestingly, these lineages appeared to have a limited distribution, which may suggest that the interplay between black-backed jackals and domestic dogs is important for establishing widespread dog-jackal rabies lineages.

Within BBJ-DD I, 14 groupings with posterior probabilities of 1.0 (except BBJ-DD I SI and SV with posterior probabilities of 0.90 and 0.97, respectively) that consisted exclusively of sequences from domestic dog cases were identified; however, the majority consisted of a limited number of sequences from a localized area in the same year and likely represented local transmission events. Six groupings contained cases that spanned across years and mapping suggested a wide dispersion of cases. The tight clustering of domestic dog cases that would suggest dog-to-dog transmission was observed in BBJ-DD I SI (Mangaung metropolitan municipality and Xhariep and Thabo Mofutsanyana districts in the Free State), BBJ-DD I SII (eThekwini metropolitan municipality in KwaZulu Natal), BBJ-DD I SIII (Ugu district in KwaZulu Natal), BBJ-DD I SIV (eThekwini metropolitan municipality and uMgungundlovu district in KwaZulu Natal), and BBJ-DD I SVI (King Cetshwayo district in KwaZulu Natal). Importantly, for BBJ-DD I SI, multiple clusters of rabies cases that are widespread along the border with Lesotho can be observed, which may suggest that black-backed jackals from Lesotho contribute to the introduction of RABV into the Free State, resulting in localized clusters of dog-to-dog transmission along the shared border. However, RABV sequence data for wildlife hosts in Lesotho are not available, and should be the focus of future studies to investigate the occurrence of the sylvatic cycle and the potential for cross-border transmission due to the natural movement of wildlife across political borders.

**Figure 8:**
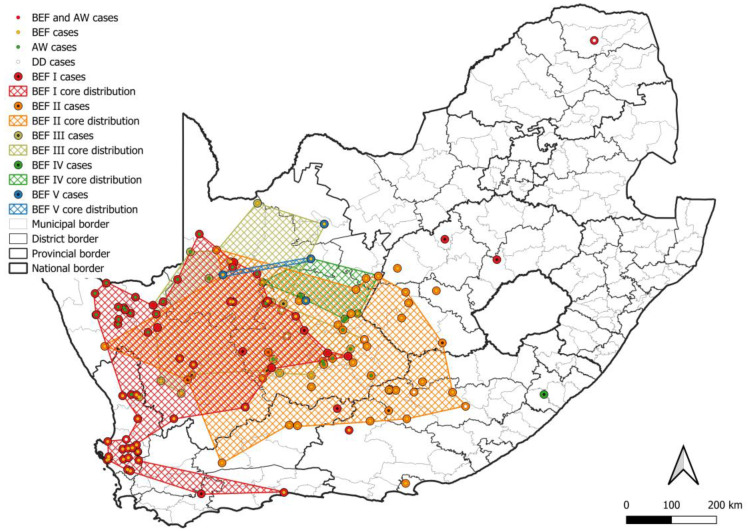
The distribution of RABV lineages maintained by bat-eared foxes in South Africa. The cases that belong to each BEF lineage are plotted. Cases reported in the dominant maintenance hosts are indicated. The map was constructed using QGIS and layered with the national, provincial, district, and municipal borders in South Africa available at https://data.humdata.org/dataset/cod-ab-zaf? (accessed on 13 April 2023).

**Figure 9:**
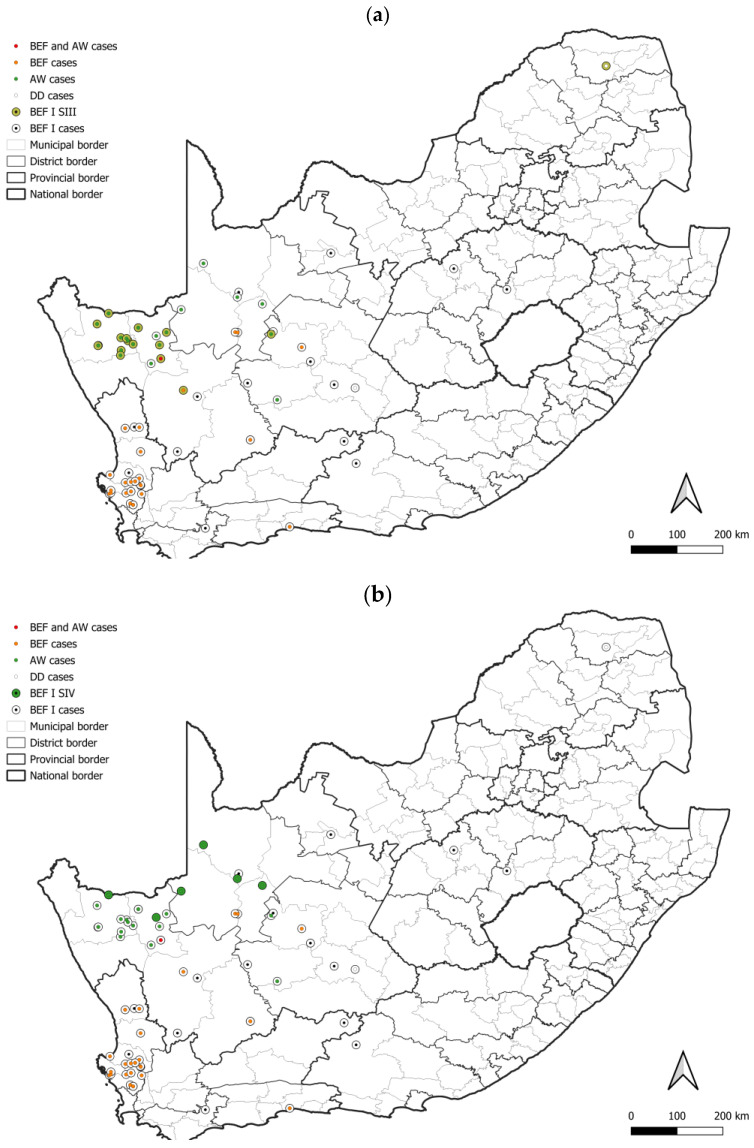
The distribution of rabies cases that belong to two groupings that consist exclusively of aardwolf cases in the BEF I lineage. All cases within the BEF I lineage are plotted and the distribution cases that belong to the (**a**) BEF I SIII, and (**b**) BEF I SIV groupings indicated. The maps were constructed using QGIS and layered with the national, provincial, district, and municipal borders in South Africa available at https://data.humdata.org/dataset/cod-ab-zaf? (accessed on 13 April 2023).

At least five lineages that appeared to be maintained by bat-eared foxes (BEF I–V) were identified, of which at least four, BEF I–BEF IV, involved the aardwolf (Figure 8). All BEF lineages were supported by bootstrap values higher than 85.0 (Table S1). The minimum distribution of all bat-eared fox lineages was partly or completely mapped to the Northern Cape province. The two most extensive bat-eared fox lineages, BEF I and BEF II, spanned into the Western Cape, and Free State and Eastern Cape provinces, respectively. Within BEF I, a host shift is evident with adaptation occurring over decades. It is important to note that based on the distribution of cases, aardwolves only play a role in RABV maintenance in fairly localized areas with little to no involvement of bat-eared foxes (Figure 9a,b). In addition, because aardwolf rabies cases can be difficult to identify and confirm, the lack of aardwolf cases in a bat-eared fox lineage may not exclude the possibility that the aardwolf plays a role in maintaining RABV, particularly in areas that are conducive to the aardwolf.

### 3.3. Rabies Control

Rabies control can be affected by the cross-border transmission of RABV either by the importation of infected animals accompanying humans or the natural movement of animals across political borders, including provincial and national borders. Phylogenetic analysis suggested that multiple RABV lineages span across national borders with neighboring countries, which included MON IV with Botswana, DD III with Eswatini, DD IV with Mozambique, BBJ-DD I with Lesotho, BBJ-DD III with Zimbabwe, and BBJ-DD V with Zimbabwe (Table S2).

A single RABV sequence from Namibia obtained from a common genet (Genetta genetta) grouped within the MON II lineage, but appeared to be geographically separated and therefore likely represents an exported case. While DD III was shared between the northeastern parts of Mpumalanga, northern parts of KwaZulu Natal, and Eswatini, it may extend to include parts of Mozambique. Due to the limited availability of RABV sequence data for some neighboring countries, additional shared lineages may exist. Coordinated, collaborative efforts may be required to establish rabies control in these regions.

The movement of RABV-infected animals poses a challenge to rabies control in South Africa. In 2010–2011, an outbreak of rabies was reported in Gauteng province due to an imported rabies case and resulted in local dog-to-dog transmission being reported from the Gauteng province for the first and only time in history [36]. Our analysis suggested that at least five independent introductions occurred from at least three RABV lineages during this outbreak, which included at least one introduction from MON I, two introductions from DD III (one likely from the Ehlanzeni district, Mpumalanga, and one likely from the uMkhanyakude district, KwaZulu Natal), and two introductions from BBJ-DD I (one from northern KwaZulu Natal and one likely from the eThekwini metropolitan municipality, KwaZulu Natal). The majority of RABV sequences during this outbreak grouped within the BBJ-DD I lineage and the introduction from northern KwaZulu Natal appeared to be the introduction that resulted in the outbreak, whereas the other introductions appeared to have resulted in dead-end infections.

Wildlife reservoirs harboring RABV without any intervention complicates rabies control. In 2016, RABV-infected black-backed jackals from the North West province introduced RABV into the West Rand district and the City of Tshwane metropolitan municipality in Gauteng, which resulted in at least 30 identified rabies cases in the black-backed jackal species. Molecular epidemiological analysis of the outbreak suggested that at least two independent introductions had occurred [33]. Our analysis suggests that cases from this outbreak could be linked to at least four RABV lineages, BBJ-DD I, BBJ-DD II, BBJ-DD III, and BBJ-DD VII, with the majority of cases linked to the BBJ-DD I lineage and cases linked to the BBJ-DD VII lineage only being reported from the City of Tshwane. Curiously, a wildlife outbreak was reported in KwaZulu Natal province around the same time and it was suggested that this outbreak resulted after spillover from domestic dogs in 2012 in the uThukela district [37]. Surveillance data suggested that at least 40 cases were identified in wildlife between 2015 and 2016 in the uMgungundlovu district, of which 24 cases were reported in black-backed jackals. Our analysis revealed that all cases were linked to the BBJ-DD I lineage. Considering the wide dispersion of cases and spillover to various wildlife species, the black-backed jackal was likely responsible for the dissemination of RABV. Kruger National Park, the largest game reserve in South Africa, reports sporadic rabies cases, primarily in infected domestic dogs that wander into the park. Our results suggest that, although rare, cases in the Kruger National Park are introduced from at least four RABV lineages, DD I, DD III, BBJ-DD II, and BBJ-DD III, which surround the park, and that cases only occurred in years in which rabies in domestic dogs were poorly controlled in areas adjacent to the park, in either Mpumalanga and/or Limpopo province. This highlights the need to prioritize the areas surrounding the park for domestic dog vaccination to prevent infected domestic dogs from entering the park and limiting the risk of spillover to wildlife species.

## 4. Discussion

In South Africa, by law, dog and cat owners are required to vaccinate their pets against rabies (The Animal Diseases Act, Act 35 of 1984) according to a prescribed schedule. Protective immunity (RABV-neutralizing antibody titers > 0.5 IU/mL) can be detected in RABV-naïve dogs 14 days after the administration of an inactivated rabies vaccine [38]. If the animal is exposed to RABV during this time, it may still become infected and potentially transmit RABV to other animals and humans. Importantly, animals with waning immunity after vaccination (RABV-neutralizing antibody titers < 0.5 IU/mL) can also become infected with RABV [38]. Taken together, this highlights (1) the need to maintain the rabies vaccination status of animals to sustain herd immunity against RABV and limit the spread of disease; (2) the need to promptly initiate vaccination campaigns at the earliest signs of an outbreak, especially in areas with low vaccination coverage, to allow sufficient time for protective immunity to be acquired, limit the rate of transmission, and ultimately disrupt the transmission cycle; and (3) the need to improve vaccination coverage in endemic regions, especially regions wherein RABV is maintained by domestic dogs, since these regions appear to be more prone to large-scale outbreaks.

**Figure 10:**
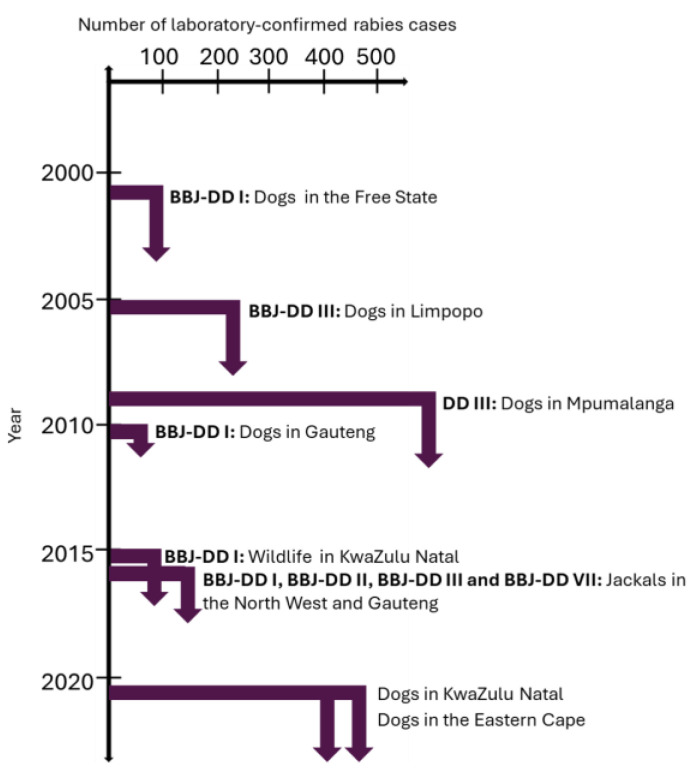
Notable outbreaks of animal rabies in South Africa and the primary lineage(s) to which the rabies cases were linked.

From 1993 to 2021, the KwaZulu Natal and Eastern Cape provinces reported the highest and second highest number of animal rabies cases, respectively, which is reflected in the number of human rabies cases reported from the two provinces [39,40]. Ongoing animal vaccination campaigns are logistically difficult to sustain and are subsequently more frequently initiated in response to an outbreak rather than proactively to prevent an outbreak. In areas where the number of animal rabies cases remains high with time, continuous rabies control efforts are required to control RABV rather than a response to developing or existing outbreaks. In 2007, a targeted vaccination program guided by new and existing data combined with rabies education via various platforms, and improved awareness and compliance with rabies surveillance and diagnostic requirements, proved to significantly reduce dog rabies cases and resulted in zero human rabies deaths in 2014 [41]. This rabies control strategy resulted in the lowest number of animal rabies cases reported from the KwaZulu Natal province in 29 years (Figure 1). In 2021, a surge in animal rabies cases occurred for the Eastern Cape and KwaZulu Natal provinces and was ascribed to two independent outbreaks of an unprecedented nature. A timeline with notable outbreaks of animal rabies in South Africa is provided in Figure 10.

Dog vaccination should be prioritized; however, accurate dog population estimates are required for planning vaccination campaigns and to ensure that at least 70% of domestic dogs are vaccinated. Importantly, 70% vaccination coverage of the entire dog population may not be required to eliminate dog-mediated human rabies but well-executed, targeted, and consistent vaccination campaigns that achieve adequate vaccination coverage, particularly in areas where RABV is maintained by domestic dogs, will likely result in the elimination of dog-mediated human rabies. While surveillance data are essential to determine patterns of disease and high-risk areas, molecular epidemiological data can provide a more complete epidemiological picture and may aid in informing the most appropriate control strategy. Importantly, inadequate or biased surveillance data can be misleading and result in crucial resources being wasted or the lack of a response when one is required. In this study, at least five lineages that appeared to be exclusively maintained by domestic dog cycles were identified in parts of the KwaZulu Natal, the Eastern Cape, and Mpumalanga provinces. Groupings that contained cases from domestic dogs only were identified in the BBJ-DD I lineage, and the mapping of the rabies cases within these groupings suggested that the black-backed jackal may play a role in their maintenance areas, with evident clustering of cases suggestive of dog-to-dog transmission. It is therefore possible that under the correct conditions, areas with dog-to-dog transmission can exist in dog-jackal lineages. In South Africa, the high-risk rabies areas typically have a high turnover of the domestic dog population and a high proportion of free-roaming dogs, owned and unowned [42,43,44]. Taken together, this highlights the importance of the domestic dog in the maintenance of RABV, particularly on the eastern side of South Africa, and the need for continued vaccination efforts in these areas.

The transport of infected animals also poses a significant risk, particularly when animals are transported to low-risk areas with inadequate vaccination coverage. Our results suggested that while the majority of introductions that occurred during an outbreak in the Gauteng province in 2010–2011 appeared to have resulted in dead-end infections or limited spillover to other animals, a single introduction under the correct circumstances may have the potential to elicit an outbreak with local dog-to-dog transmission in an area that was otherwise considered to be free from rabies. Evidence of multiple introductions into the Gauteng province during this outbreak is likely the result of enhanced awareness and is a cause for concern since frequent introductions of RABV-infected animals into a province that is highly populated, is non-endemic for dog rabies, and likely has inadequate dog vaccination rates to prevent a rabies outbreak in domestic dogs can result in a large-scale rabies outbreak with potential human exposure to RABV.

In South Africa, the number of rabies cases reported in wildlife is low compared to that in domestic dogs, which may be linked with the passive surveillance or inadequate surveillance of rabies in the country. However, the expansion of wildlife RABV could have significant consequences and result in spillover to domestic dogs, livestock, other wildlife, and humans. Since 2016, at least two outbreaks in wildlife wherein RABV-infected black-backed jackals appeared to have played an important role in the introduction and/or distribution of RABV into a region that did not previously report jackal rabies have been reported [33,37]. In contrast, the near disappearance of wildlife rabies cases in two provinces, Mpumalanga and the Free State provinces, where these species have been considered to be the dominant maintenance hosts, require further investigation to determine whether administrative, surveillance-related, ecological, and/or anthropogenic factors may have resulted in the low number of cases reported. A recent study that assessed rabies surveillance in South Africa based on the laboratory submission and testing data suggested that inadequate surveillance occurred in 26/52 (50.00%) of districts in South Africa between 2010 and 2019 [20]. The same study demonstrated that while surveillance is biased toward domestic animals, it is still inadequate in 20/51 (39.22%) of districts with adequate dog population densities to theoretically sustain RABV transmission, of which 8/20 (40.00%) were districts that formed part of significant rabies disease clusters in domestic animals [20]. Low wildlife-per-human testing rates (WHRs) were reported for 29 districts that are within the home range of at least one of the known wildlife maintenance hosts in South Africa [20]. The findings from this study suggested that surveillance-related factors may have contributed to the near disappearance of wildlife rabies cases in some provinces. Interestingly, adequate surveillance was demonstrated for the Free State province in both domestic animals and wildlife [20], suggesting that other factors may be contributing to the lack of rabies cases reported in the yellow mongoose from the Free State province.

The aardwolf is an emerging maintenance host in South Africa, and was recently reported to emerge due to a host shift from bat-eared foxes in the Northern Cape [7]. In this study, two groupings that consisted exclusively of RABV sequences from aardwolf rabies cases were identified, which is in agreement with previous findings [7], and appeared to have limited distribution (Figure 9a,b). It is important to note that this is only representative of the role of the aardwolf in parts of the Northern Cape and does not describe the role of the aardwolf in the Free State. With the contrasting epidemiological profiles of these two regions, the role of the aardwolf in rabies epidemiology in these two regions may differ and the role of the aardwolf in the Free State warrants investigation. RABV transmission is mainly enhanced by direct host-to-host contact, and hence a better understanding of the interaction between aardwolves and bat-eared foxes is of pivotal interest to clarify the involvement of the two host species in the epidemiology of this disease in the Northern Cape. The use of radio collars may provide some insights.

It is important to acknowledge that geographical features like mountains and rivers may impact the distribution of a lineage along with the natural habitat and behavior of the maintenance host(s), including changes in such behavior due to changes in the environment. Another important consideration, particularly for rabies cases resulting from the transportation of a RABV-infected animal to areas that are non-endemic for canine rabies, is access to the national transportation network. Cross-border transmission may complicate rabies control and can result in the repeated introduction of RABV into adjacent areas. Once rabies is under control in an area, buffer zones to limit the spread of RABV across political borders should be identified to reduce the likelihood of the spread of RABV. It is important to note that these zones will not prevent the importation of cases by humans transporting animals across established buffer zones. Buffer zones may be particularly useful in areas where wildlife hosts play a role in introducing RABV into adjacent areas. For example, the black-backed jackal has a wide home range, which is not maintained by political borders, and can result in the continued introduction of RABV into adjacent regions, particularly if the vaccination coverage falls below the protective threshold. If a buffer zone cannot be established in a region, rabies control programs should be implemented as a collaborative effort that involves all regions to improve the likelihood of successful rabies control, considering that continued spillover of RABV from high-risk areas to areas where rabies is under control presents a significant challenge to rabies control. In addition, the short-term control of rabies in areas where RABV is exclusively maintained by domestic dogs may not be sufficient to prevent dog-mediated human deaths, since spillover from wildlife maintenance hosts to naïve domestic dog populations may still occur, which will result in the reintroduction of RABV into the domestic dog population. It is therefore important to consider the potential risk in areas with wildlife RABV, which would require a high vaccination coverage in the domestic dog population to limit the risk of spillover events. In addition, future rabies control strategies would likely need to include targeted bait vaccination to disrupt wildlife RABV that poses a risk of reintroduction into the domestic dog population, particularly vaccines that target black-backed jackal populations at key points. The Raboral V-RG^®^ vaccine, a recombinant vaccinia viral vectored vaccine that encodes the RABV glycoprotein, has been used in parts of Europe in wildlife populations and was evaluated in captive and free-ranging black-backed jackals in South Africa. This study demonstrated protective antibody levels (RABV-neutralizing antibody titer > 0.5 IU/mL) after a single vaccine dose in the majority of captive black-backed jackals for up to 12 weeks, while protective antibody levels were demonstrated in fewer free-ranging black-backed jackals between 3 and 18 months, depending on the study site [45]. Importantly, this study highlighted the need for an improved understanding of the factors that may influence the uptake of bait vaccines by wildlife populations, including but not limited to the baiting strategy, target species population density, turnover, and changes that affect the population distribution, and the need for additional research to evaluate the effectiveness of bait vaccines at inducing population immunity and preventing rabies outbreaks in targeted wildlife populations [45].

The elimination of dog-mediated human rabies requires political will to drive rabies surveillance, vaccination, and education activities, which are the three pillars of successful rabies elimination programs. The continued generation and analysis of surveillance and molecular epidemiological data are invaluable to understand rabies epidemiology. It is important to acknowledge that rabies epidemiology can change with time and that continuous monitoring is required to identify changes that could have implications for rabies control. In this study, we evaluated all retrospective rabies data for South Africa available on publicly available databases, evaluated the distribution and maintenance host(s) of rabies cases based on surveillance data, and compared this with the distribution and maintenance host(s) of rabies cases that were genetically linked and grouped into different lineages. This analysis provided an improved understanding of the distribution of the different RABV lineages in South Africa, importantly identifying areas that can be targeted for rabies control strategies to limit future spread of RABV. In South Africa, resources are limited and many competing interests stretch the available resources even more. Therefore, we frequently do not have the sustained provision of committed resources, and the optimal use through targeted, well-executed vaccination campaigns is required to obtain population immunity in key areas to disrupt the cycle of transmission. The data in this study should be interpreted with the latest available surveillance data and should be updated with the availability of newly generated RABV sequence data.

## 5. Limitations of This Study

In this study, animal rabies cases in South Africa were reviewed from data available from January 1993 until December 2021, with a more detailed district-level analysis from January 2010 until December 2021. It is important to acknowledge that due to the passive surveillance of rabies in South Africa and other service and/or administrative challenges, these figures likely represent an underestimation of the actual rabies number of cases in South Africa and only reflect the cases available on the DALRRD database for animal diseases in South Africa. It is important to acknowledge that not all animals that succumb to rabies are submitted for rabies testing. Wildlife rabies cases are particularly difficult to identify and confirm, and may be highly underestimated. The cases in this report only represent laboratory-confirmed cases of animal rabies reported to DALRRD and rely on the reported species identification.

In addition, reporting bias likely exists due to the nature of surveillance and the location of rabies reference laboratories, with regions closer to the reference laboratories being better represented than those that are far from the reference laboratories. South Africa has two reference laboratories for the confirmation of animal rabies cases, which include the Agricultural Research Council-Onderstepoort Veterinary Institute (ARC-OVI) in Pretoria, Gauteng, and the Allerton Provincial Veterinary Laboratory in Pietermaritzburg, KwaZulu Natal.

The mapping of cases relies on the accurate capture of coordinates. However, the coordinates are frequently not reported and the coordinates of the closest city/town/farm are subsequently used. The lineages reported in this study are based on the current available cytoplasmic domain of the glycoprotein and G-L intergenic sequence data, which may not represent all the RABV lineages or reflect the true distribution of lineages in South Africa since limited sequence data were available for some RABV lineages, geographical regions, and animal species. Additional sequence data from underrepresented areas and animal species are required to allow more accurate distribution mapping. In addition, the sequence data available are not from systematically sequenced samples but from various studies with specific research questions and from outbreak investigations, which introduces selection bias.

## Data Availability

All relevant data are included within the manuscript and its Supplementary Materials.

## Supplementary Materials

The following supporting information can be downloaded at: https://www.mdpi.com/article/10.3390/v17030340/s1, Table S1: Number of laboratory-confirmed animal rabies cases per species, 1993–2021; Table S2: Summary of the distribution of rabies lineages in South Africa; Figure S1. Phylogenetic trees for lineages grouped based on the dominant maintenance host/s identified in the lineage. ML phylogenetic trees annotated with the lineages identified with the same dominant maintenance host/s, including the (a) MON, (b) DD, (c) BBJ-DD, and (d) BEF lineages; Phylotree S1: MON lineages; Phylotree S2: DD lineages; Phylotree S3: BBJ-DD lineages; Phylotree S4: BEF lineages; Phylotree S5: Complete ML tree; Phylotree S6: BEAST tree for MON lineages; Phylotree S7: BEAST tree for DD lineages; Phylotree S8: BEAST tree for BBJ-DD lineages; Phylotree S9: BEAST tree for BEF lineages; Phylotree S10: BEAST tree for BBJ-DD I lineage.
