# Supplementary material for: Temporal and Spatial Analysis of Rabies Virus Lineages in South Africa
Source: Viruses. 2025 Feb 28;17(3):340. doi: 10.3390/v17030340 (PMC11946777; doi:10.3390/v17030340)
Supplement: Supplementary file 1 [file viruses-17-00340-s001.zip › 08_02_2025_Viljoen_Suppl material.pdf]

Supplementary material

Supplementary tables

Table S1: Number of laboratory-confirmed animal rabies cases per species, 1993-2021.

**Species**<br>**Scientific name**	**Number of cases (%)**	**Species**<br>**Scientific name**	**Number of cases (%)**	**Species**<br>**Scientific name**	**Number of cases (%)**
Aardwolf<br>*Proteles cristata*	98<br>(0.76%)	Caracal<br>*Caracal caracal*	9<br>(0.07%)	Selous' mongoose<br>*Paracynictis selousi*	2<br>(0.02%)
African buffalo<br>*Syncerus caffer*	1<br>(0.01%)	Chacma baboon<br>*Papio ursinus*	1<br>(0.01%)	Serval<br>*Leptailurus serval*	4<br>(0.03%)
African civet<br>*Civettictis civetta*	7<br>(0.05%)	Common duiker<br>*Sylvicapra grimmia*	10<br>(0.08%)	Side-striped jackal<br>*Canis adustus*	18<br>(0.14%)
African wildcat<br>*Felis lybica*	16<br>(0.12%)	Dwarf mongoose<br>*Helogale parvula*	1<br>(0.01%)	Slender mongoose<br>*Galerella sanguinea*	68<br>(0.53%)
Avian<br>*Aves*	1<br>(0.01%)	Eland<br>*Taurotragus oryx*	3<br>(0.02%)	Small grey mongoose<br>*Herpestes pulverulentus*	26<br>(0.20%)
Banded mongoose<br>*Mungos mungo*	2<br>(0.02%)	Equine<br>*Equus caballus*	83<br>(0.64%)	Small spotted cat<br>*Felis nigripes*	5<br>(0.04%)
Bat-eared fox<br>*Otocyon megalotis*	409<br>(3.18%)	Unspecified farm animal<br>Species not indicated	35<br>(0.27%)	Small spotted genet<br>*Genetta genetta*	28<br>(0.22%)
Black-backed jackal<br>*Canis mesomelas*	301<br>(2.34%)	Feline<br>*Felis catus*	413<br>(3.21%)	Spotted hyena<br>*Crocuta crocuta*	4<br>(0.03%)
Blue wildebeest<br>*Connochaetes taurinus*	1<br>(0.01%)	Honey badger<br>*Mellivora capensis*	15<br>(0.12%)	Steenbok<br>*Raphicerus campestris*	2<br>(0.02%)
Bovine<br>*Bos taurus*	1985<br>(15.41%)	Kudu<br>*Tragelaphus strepsiceros*	8<br>(0.06%)	Striped polecat<br>*Ictonyx striatus*	14<br>(0.11%)
Brown hyena<br>*Parahyaena brunnea*	1<br>(0.01%)	Large grey genet<br>*Genetta*	1<br>(0.01%)	Pig<br>*Sus scrofa domesticus*	21<br>(0.16%)
Burchell's zebra<br>*Equus quagga burchellii*	2<br>(0.02%)	Large grey mongoose<br>*Herpestes ichneumon*	27<br>(0.21%)	Suricate<br>*Suricata suricatta*	116<br>(0.90%)
Canine<br>*Canis lupus familiaris*	7914<br>(61.45%)	Lion<br>*Panthera leo*	6<br>(0.05%)	Viverrids<br>*Viverridae*	3<br>(0.02%)
Cape clawless otter<br>*Aonyx capensis*	2<br>(0.02%)	Ostrich<br>*Struthio camelus*	1<br>(0.01%)	Water mongoose<br>*Atilax paludinosus*	33<br>(0.26%)
Cape fox<br>*Vulpes chama*	21<br>(0.16%)	Ovine<br>*Ovis aries*	171<br>(1.33%)	White-tailed mongoose<br>*Ichneumia albicauda*	10<br>(0.08%)
Cape ground squirrel<br>*Xerus inauris*	16<br>(0.12%)	Rock hyrax (dassie)<br>*Procavia capensis*	6<br>(0.05%)	African wild dog<br>*Lycaon pictus*	5<br>(0.04%)
Caprine<br>*Capra aegagrus hircus*	277<br>(2.15%)	Rodent<br>*Rodentia*	1<br>(0.01%)	Yellow mongoose<br>*Cynictis penicillata*	675<br>(5.24%)

Table S2: Summary of the distribution of rabies lineages in South Africa.

Lineage (bootstrap support):	Province:	District/metropolitan municipality:	Possible extended distribution:	Neighboring country:
MON I (99.7)	Free State	Fezile Dabi	Francis Baard, Northern Cape<br>Reason: Parts of this district are included in the distribution of the cycle after mapping cases that belong to this cycle	
		Lejweleputswa		
		Thabo Mofutsanyana		
	Gauteng	West Rand		
		Sedibeng		
	Mpumalanga	Gert Sibande		
	Northern Cape	Pixley ka Seme		
	North West	Dr Kenneth Kaunda		
		Dr. Ruth Segomotsi Mompati		
		Ngaka Modiri Molema		
MON II (100.0)	Eastern Cape	Chris Hani	Central Karoo, Western Cape and Nelson Mandela Bay, Eastern Cape<br>Reason: Limited sequence data for the district and metropolitan municipality<br><br>Amathole, Eastern Cape<br>Reason: Parts of this district are included in the distribution of the cycle after mapping cases that belong to this cycle	
		Joe Gqabi		
		Sarah Baartman		
	Free State	Lejweleputswa		
		Mangaung		
		Thabo Mofutsanyana		
		Xhariep		
	Northern Cape	Francis Baard		
		Pixley ka Seme		
MON III (56.5)	Mpumalanga	Gert Sibande		
		Nkagala		
MON IV (100.0)	Northern Cape	John Taolo Gaetsewe	Pixley ka Seme, Northern Cape<br>Reason: Parts of this district are included in the distribution of the cycle after mapping cases that belong to this cycle	Botswana
		ZF Mgcawu		
	Western Cape	West Coast		
DD I (97.6)	Eastern Cape	Amathole	Alfred Nzo, Eastern Cape and parts of southern KwaZulu Natal<br>Reason: Evidence of sporadic cases	
		Chris Hani		
		Joe Gqabi		
		OR Tambo		
DD II (98.8)	Eastern Cape	Alfred Nzo		
		Amathole		
		Chris Hani		
		Joe Gqabi		
		OR Tambo		
	KwaZulu Natal	eThekwini		
		Harry Gwala		
		Ugu		
DD III (81.0)	KwaZulu Natal	uMkhanyakude		Eswatini
		Zululand		
	Mpumalanga	Ehlanzeni		
		Gert Sibande		
	Limpopo	Mopani		
DD IV (99.1)	Mpumalanga	Ehlanzeni		Mozambique
DD V (74.9)	KwaZulu Natal	Zululand		
	Mpumalanga	Gert Sibande		
	Eastern Cape	Chris Hani		Lesotho

BBJ-DD I (85.5)		Joe Gqabi	Parts of Alfred Nzo and OR Tambo, Eastern Cape, and parts of Fezile Dabi and Lejweleputswa, Free State<br>Reason: Parts of these districts are included in the distribution of the cycle after mapping cases that belong to this cycle	
	Free State	Mangaung		
		Thabo Mofutsanyana		
		Xhariep		
	Gauteng	City of Tshwane		
	KwaZulu Natal	All districts and eThekwini		
	Limpopo	Waterberg		
	Mpumalanga	Gert Sibande		
	North West	Bojanala Platinum		
		Ngaka Modiri Molema		
		Dr Kenneth Kaunda		
BBJ-DD II (95.7)	North West	Dr. Ruth Segomotsi Mompati		
		Ngaka Modiri Molema		
BBJ-DD III (91.2)	Limpopo	Mopani	Capricorn, Limpopo<br>Reason: Parts of these districts are included in the distribution of the cycle after mapping cases that belong to this cycle	Zimbabwe
		Vhembe		
		Waterberg		
	Mpumalanga	Ehlanzeni		
BBJ-DD IV (93.5)	Limpopo	Vhembe	Capricorn, Limpopo<br>Reason: Parts of these districts are included in the distribution of the cycle after mapping cases that belong to this cycle	
		Waterberg		
	North West	Bojanala Platinum		
BBJ-DD V (97.2)	Limpopo	Capricorn		Zimbabwe
		Vhembe		
		Waterberg		
BBJ-DD VI (99.9)	Limpopo	Waterberg		
BBJ-DD VII (86.4)	Gauteng	City of Tshwane		
BEF I (97.9)	Northern Cape	Namakwa	Central Karoo, Western Cape, and Sarah Baartman, Eastern Cape<br>Reason: Limited sequence data for these districts	
		Pixley ka Seme		
		ZF Mgcawu		
	Western Cape	Garden Route		
		Overberg		
		West Coast		
BEF II (96.2)	Eastern Cape	Chris Hani	Joe Gqabi and Sarah Baartman, Eastern Cape and<br>Lejweleputswa and Mangaung, Free State<br>Reason: Isolated cases outside the core distribution	
	Free State	Xhariep		
	Northern Cape	Francis Baard		
		Pixley ka Seme		
		Namakwa		
		ZF Mgcawu		
	Western Cape	Central Karoo		
BEF III (98.8)	Northern Cape	John Taolo Gaetsewe	West Coast, Western Cape<br>Reason: Isolated case outside the core distribution	
		Namakwa		
		Pixley ka Seme		
		ZF Mgcawu		
BEF IV (86.1)	Northern Cape	Francis Baard		
		Pixley ka Seme		
		ZF Mgcawu		
BEF V (98.1)	Northern Cape	ZF Mgcawu		

Supplementary figures

a) MON lineages

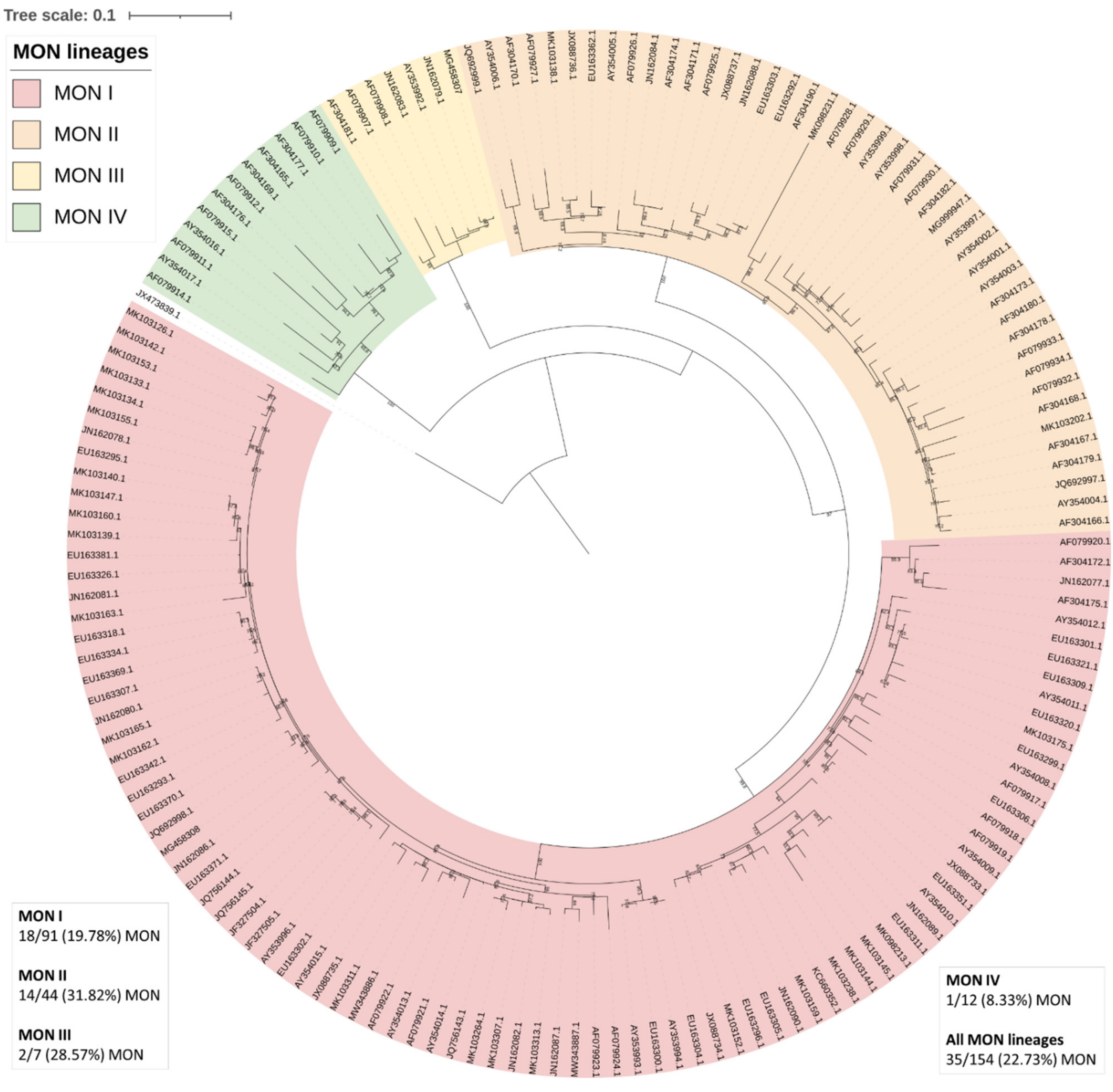

b) DD lineages

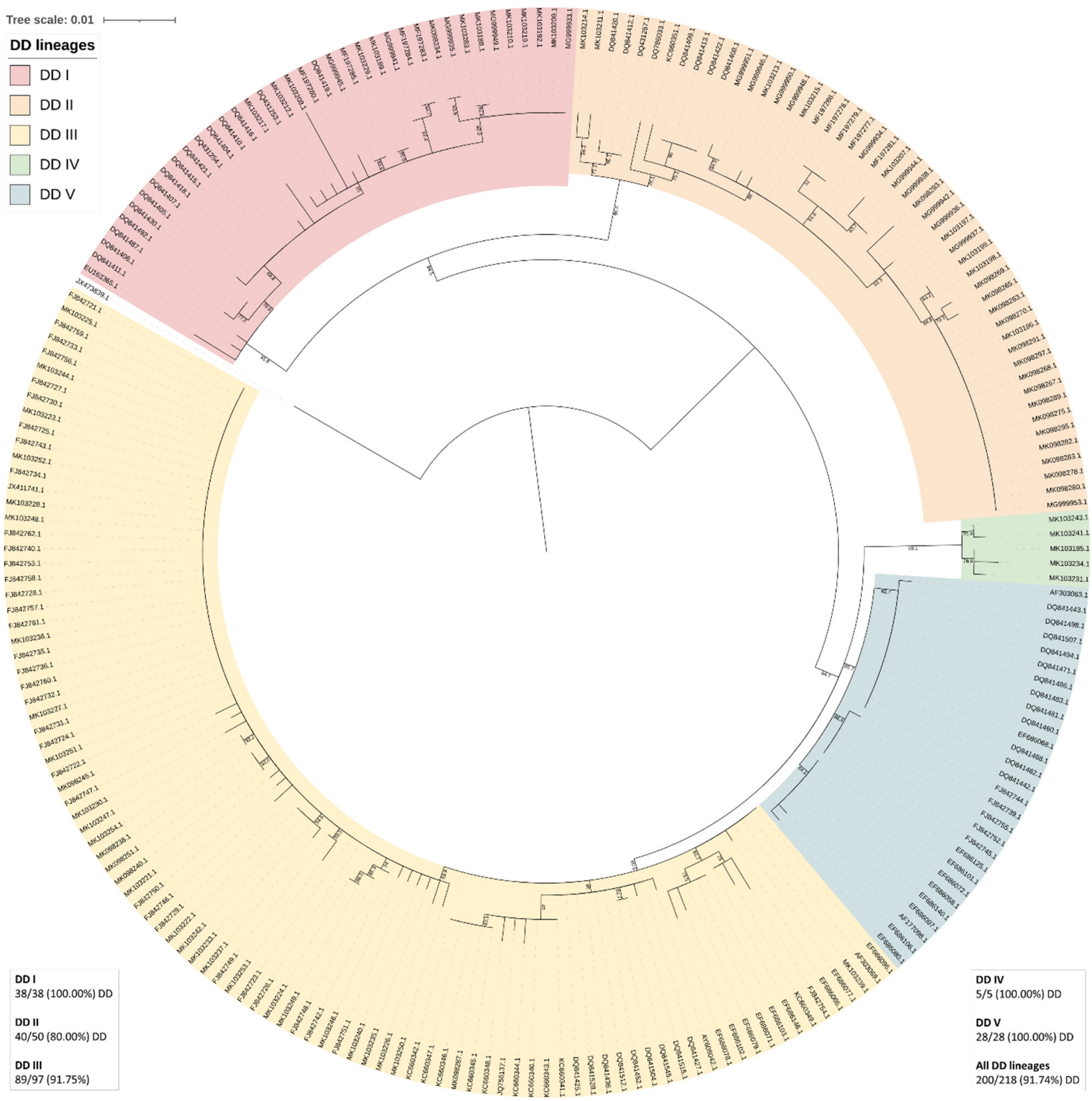

c) BBJ-DD lineages

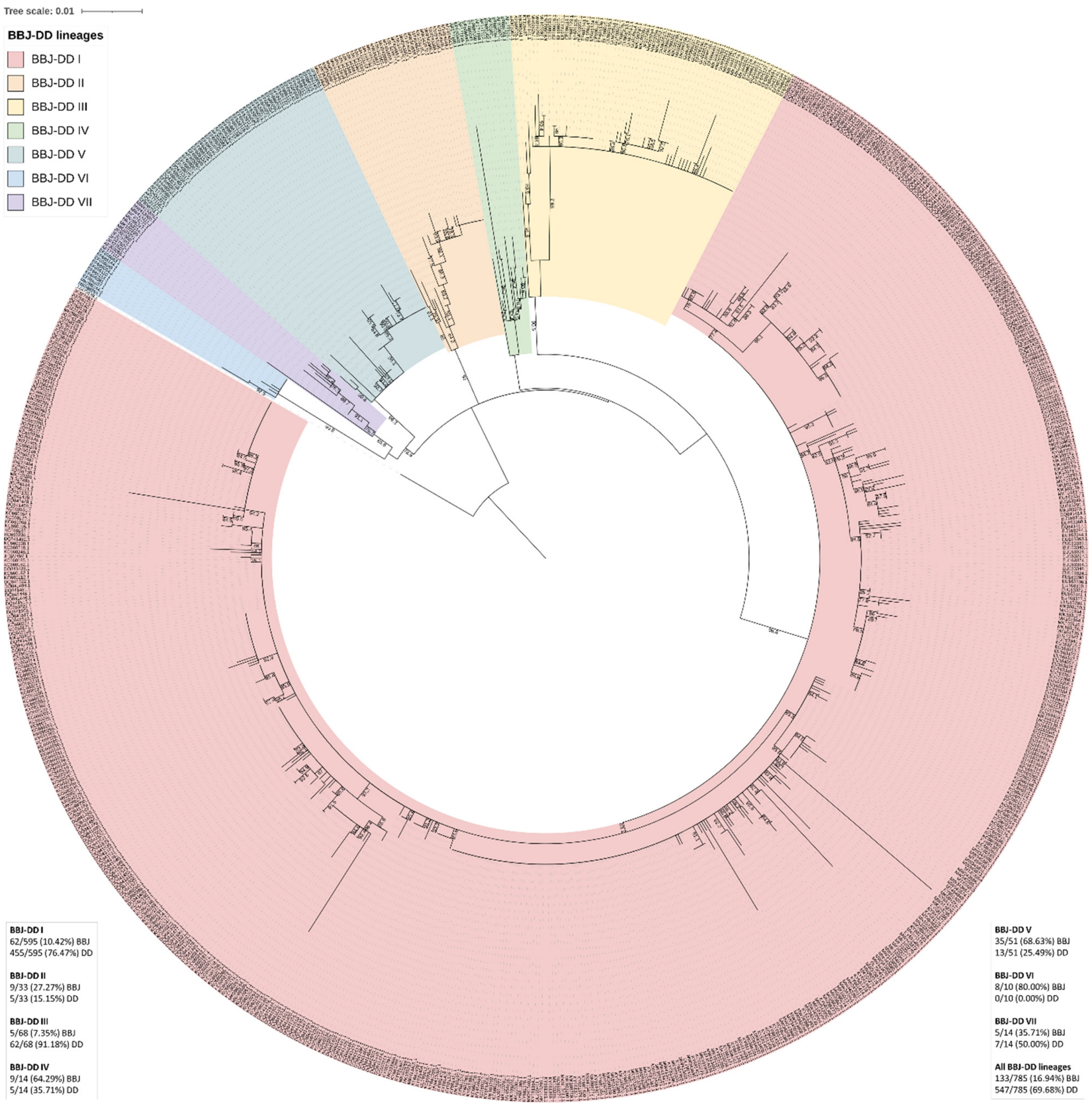

d) BEF lineages

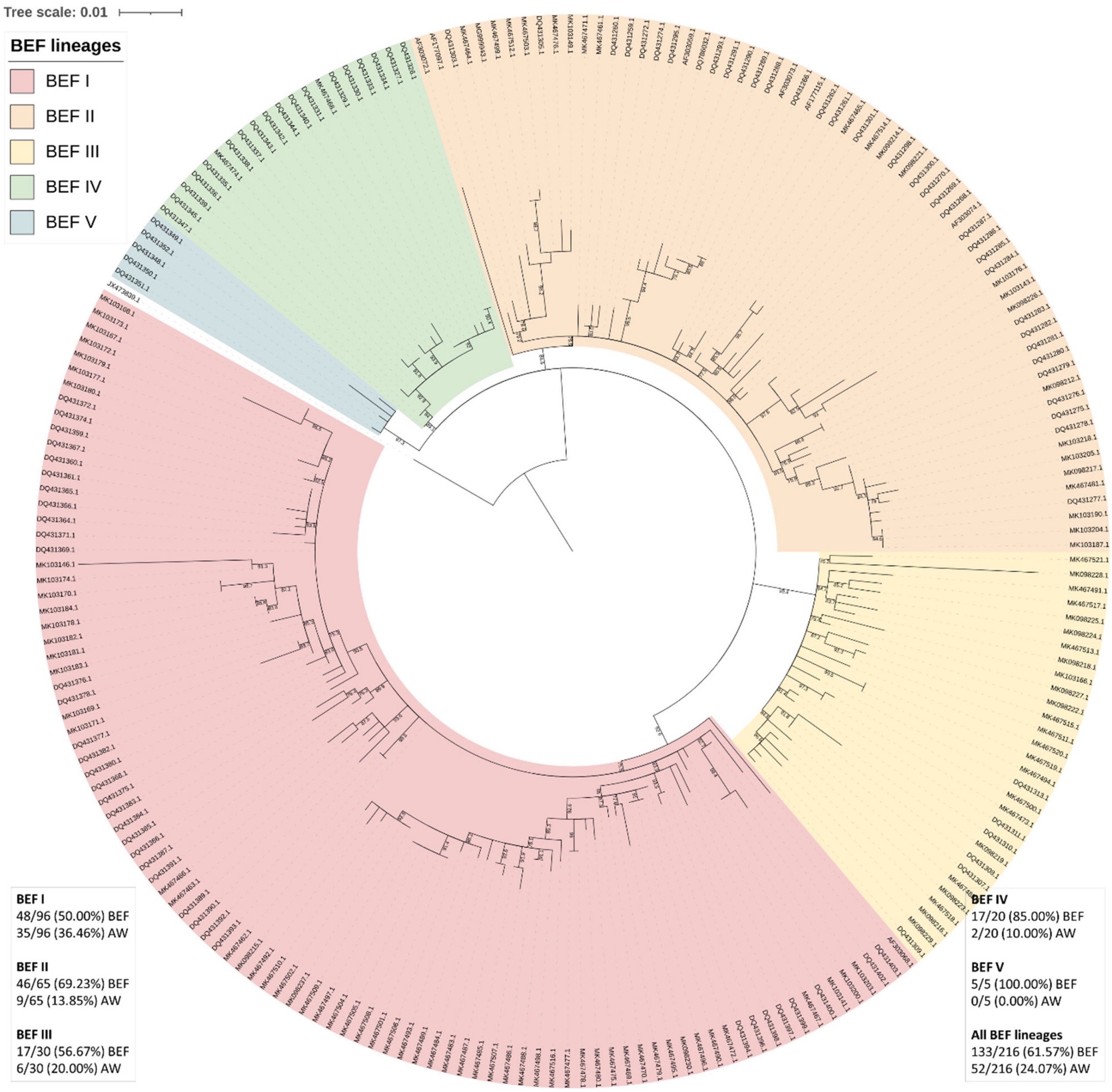

Figure S1. Phylogenetic trees for lineages grouped based on the dominant maintenance host(s) identified in the lineage. ML phylogenetic trees annotated with the lineages identified with the same dominant maintenance host(s), including the a) MON, b) DD, c) BBJ-DD, and d) BEF lineages. The host distribution for the maintenance host(s) that appear to be important for maintenance is indicated for each lineage and the combined total for all lineages with the same maintenance host(s).
