# Supplementary figures and images for: Temporal and Spatial Analysis of Rabies Virus Lineages in South Africa
Source: Viruses. 2025 Feb 28;17(3):340. doi: 10.3390/v17030340 (PMC11946777; doi:10.3390/v17030340)

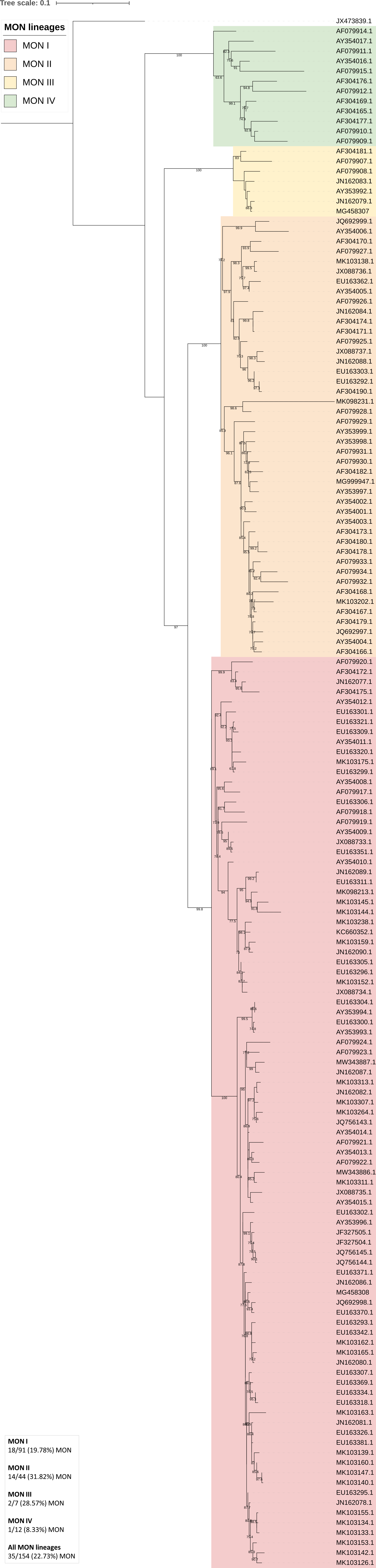

Supplement: Supplementary file 1 [file viruses-17-00340-s001.zip › Phylotree S1_MON.tif]

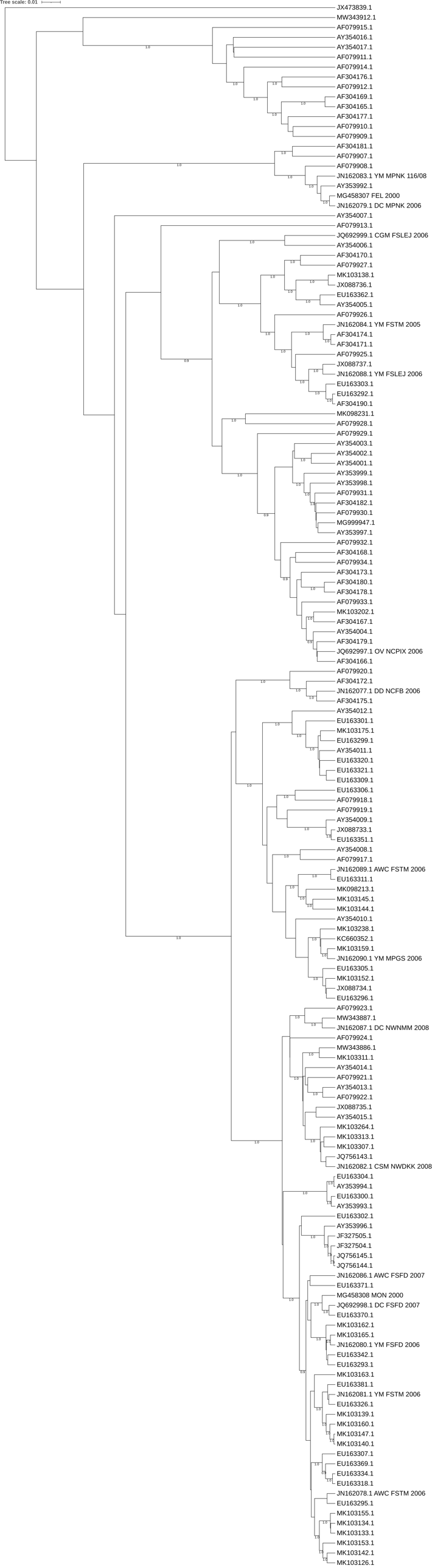

Supplement: Supplementary file 1 [file viruses-17-00340-s001.zip › Phylotree S6_ BEAST tree for MON lineages.tif]

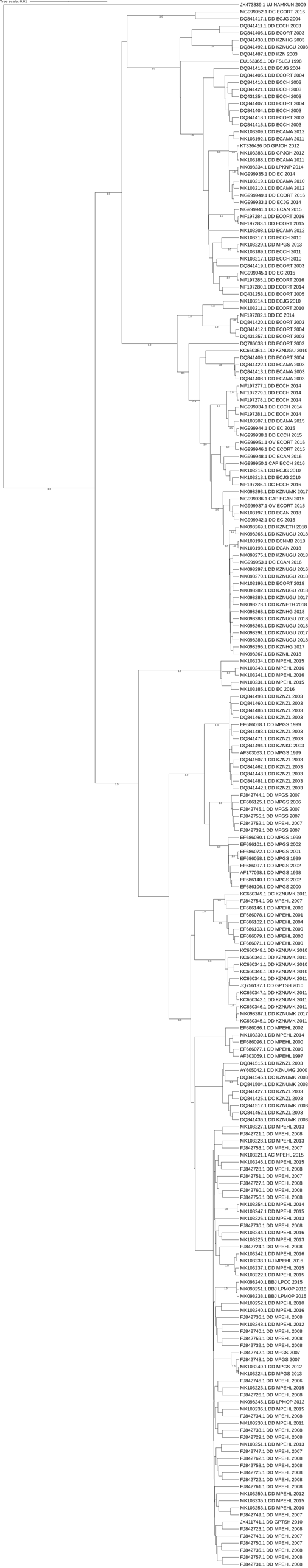

Supplement: Supplementary file 1 [file viruses-17-00340-s001.zip › Phylotree S7_BEAST tree for DD lineages.tif]

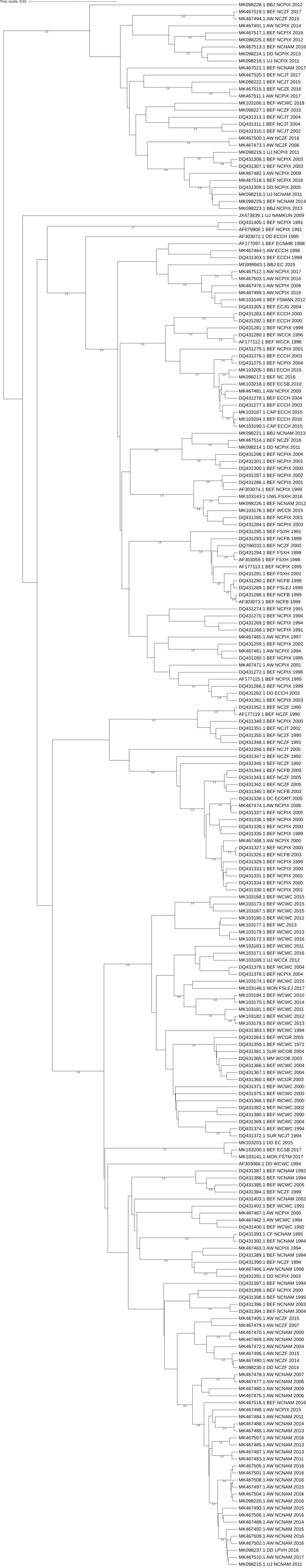

Supplement: Supplementary file 1 [file viruses-17-00340-s001.zip › Phylotree S9_BEAST tree for BEF lineages.tif]
